# Supplementary material for: Learning curve and surgical outcome of robotic assisted colorectal surgery with ERAS program
Source: Sci Rep. 2022 Nov 29;12:20566. doi: 10.1038/s41598-022-24665-w (PMC9709162; doi:10.1038/s41598-022-24665-w)
Supplement: Supplementary file 1 — Supplementary Information 1. [file 41598_2022_24665_MOESM1_ESM.zip › LSC Raw data-Ñ[▒K/085/N_ñw╝╨░O▒Kñσ.pdf]

W55 / W55

臺中榮民總醫院
手術室護理記錄

[redacted]

基本資料

- 室別： 開刀房-06室，手術科別： CRS，手術類別： 常規，日期： 2020/07/22
- 進入時間： 0825 ； 麻醉開始時間： 0830 ； Time Out時間： 0920 ； 劃刀時間： 0921 ； 手術結束時間： 1350 送出時間： 1400 ；
- 麻醉方式： GE
  診斷：S colon ca s/p loop T
  手術名稱：Laparoscopic Anterior resection and anastomosis - malignant
  手術局部用藥：nil
  術前預防性抗生素：
  - 抗生素名稱一： Veterin 1GM IVD麻醉護理師給予
  - 時間： 0855
  - 上次簽章: 朱芸萱

  ，追加預防性抗生素：
  - 抗生素名稱一： Veterin 1GM IVD麻醉護理師給予
  - 時間： 1255
  - 上次簽章: 張湘玲
- 手術醫師： 林俊余，林昌霖，蔡濬樟
- 麻醉醫師： Fellow 楊上杰
-
  - 刷手護士A ： 張湘玲，時間： 0825，接替刷手一： 林愛甯，時間： 1200
  - 流動護士A ： 朱芸萱，時間： 0825，接替流動一： 張湘玲，時間： 1230

皮膚狀況

A-V Shunt： 無
術前： 完整
記錄者： 上次簽章: 朱芸萱
術後： 完整
記錄者： 上次簽章: 張湘玲

手術準備

手術臥式 單一手術臥式

-
  - 手術臥式： 截石臥式
  - 使用附件： 溫毯 43 ℃，頭圈，腳架
  - 皮膚準備： 10%Aq Betadine，Hibiscrub，2% Chlorhexidine
  - 電燒： 使用電燒: 電極片位置： 其它： 迴路電極墊，記錄者： 上次簽章: 朱芸萱

止血帶T： 無

植入物

- 植入物名稱/號碼/數量/部位：ETHICON ENDO-GIA 60-4.1mm/３/腸段

，記錄者： 上次簽章: 朱芸萱

標本送檢

- 細菌培養 無
- 細胞學 無
- 冰凍切片 無
- 病理標本
  組織名稱一：D-colon
  ，
  組織名稱二：L't colic root
- 截肢 無

標本交付 標本： 2 袋 ， 標本： 0 盒 ， 標本： 0 瓶 ， 細胞學： 0 管 ， Culture Tube： 0 管 ， 被交付醫師： R 蔡濬樟 ，

- 記錄者： 上次簽章: 張湘玲
- 時間： 1330

傷口術後敷料

傷口敷料： 傷口敷料： 無

- 請於圖上標示部位：

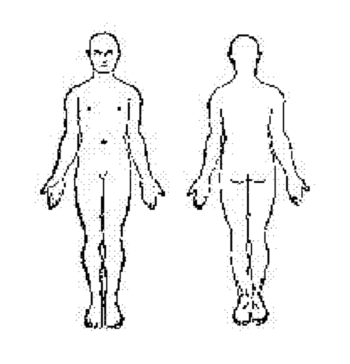

- 傷口敷料： 軟性敷料

導管

導管	天數	導管資訊	插入日期	插入時間	更換日期	拔除日期	拔除時間	拔除原因	預計拔除/更換	預計拔除/更換日期
Foley(1)	1	號碼:16 Fr.; 部位:膀胱;	2020/07/22	0845						
A-line (1)	1	號碼:20 G; 部位:右手;	2020/07/22	0845						
Sump NG Tube(1)	1	號碼:16 Fr.; 深度:55cm; 部位:胃;	2020/07/22	0855						

用物計數

- 手術中出血量： 少 c.c.
- 手術中尿量： 150 c.c.
-
    - 其它： 無

用物計數： 多切口同時縫合

- 1.器械： 正確 ， 2.縫針： 正確 ， 3.刀片： 正確 ， 4.紗布： 正確 ， 5.腹部墊： 未使用 ， 6.紗球： 未使用 ， 7.PEANUTS ： 未使用 ， 8.紗條： 未使用 ， 9.絲帶： 未使用 ， 10.針頭： 未使用 ， 11.Vessel loop： 未使用 ， 12.Boots： 未使用 ， 13.Jelco： 未使用 ， 14.棉條： 未使用 ，
    - 第一次：
        - 刷手護士 ： 林愛甯
        - 流動護士： 上次簽章: 張湘玲
        - 時間： 1245

  ,
    - 第二次：
        - 刷手護士 ： 林愛甯
        - 流動護士： 上次簽章: 張湘玲
        - 時間： 1255

  ,
    - 第三次：
        - 刷手護士 ： 林愛甯
        - 流動護士： 上次簽章: 張湘玲
        - 時間： 1330

備註

備註： 3D scope

- 記錄者： 上次簽章: 朱芸萱
- 時間： 0900

填寫護理人員 張湘玲

正式摘要

W55 / W55

doctorRank:B
